# Self-images in the present and future: Role of affect and the bipolar phenotype

**DOI:** 10.1016/j.jad.2015.08.042

**Published:** 2015-11-15

**Authors:** Martina Di Simplicio, Emily A. Holmes, Clare J. Rathbone

**Affiliations:** a MRC Cognition and Brain Sciences Unit, Cambridge,UK; b Department of Clinical Neuroscience, Karonlinska Institutet, Stockholm, Sweden; c Department of Psychology, Social Work and Public Health, Oxford Brookes University, Oxford, UK

**Keywords:** Hypomania, Self-image, Identity, Mood, Anxiety, Prospection, Bipolar disorder

## Abstract

**Background:**

Bipolar Spectrum Disorder (BPSD) is associated with changes in self-related processing and affect, yet the relationship between self-image and affect in the BPSD phenotype is unclear.

**Methods:**

47 young adults were assessed for hypomanic experiences (BPSD phenotype) using the Mood Disorders Questionnaire. Current and future self-images (e.g. I am… I will be…) were generated and rated for emotional valence, stability, and (for future self-images only) certainty. The relationship between self-image ratings and measures of affect (depression, anxiety and mania) were analysed in relation to the BPSD phenotype.

**Results:**

The presence of the BPSD phenotype significantly moderated the relationship between (1) affect and stability ratings for negative self-images, and (2) affect and certainty ratings for positive future self-images. Higher positivity ratings for current self-images were associated with lower depression and anxiety scores.

**Limitations:**

This was a non-clinical group of young adults sampled for hypomanic experiences, which limits the extension of the work to clinical levels of psychopathology. This study cannot address the causal relationships between affect, self-images, and BPSD. Future work should use clinical samples and experimental mood manipulation designs.

**Conclusions:**

BPSD phenotype can shape the relationship between affect and current and future self-images. This finding will guide future clinical research to elucidate BPSD vulnerability mechanisms and, consequently, the development of early interventions.

## Highlights

• BPSD phenotype moderates the relationship between affect and self-images.
• Low mood is associated with less positivity of self-images in healthy young adults.
• These findings can help inform cognitive models of bipolar psychopathology.
• These findings can guide psychological treatment innovation.

## 1 Introduction

It is increasingly recognised that the way we view ourselves (self-image) is related to our mood (Rathbone et al., 2015). Bipolar spectrum disorders (BPSD) are characterized by mood disturbances which warrant further examination at a psychological level. Very little work has directly addressed the relationship between self and affect in BPSD, or young adults in particular. Cognitive-behavioural therapy for BPSD has shown mixed outcomes so far (Thase et al., 2014), with some evidence that assumptions and beliefs about the self may moderate response to treatment (Lam et al., 2005). Onset of BPSD is typically in early adulthood (Merikangas et al., 2011), which represents an important period for development of the self (Fitzgerald, 1988; Rathbone et al., 2008, Burnett Heyes et al., 2013). Psychopathology research can contribute to treatment innovation by focusing on aspects of psychopathology that remain insufficiently explored yet (Di Simplicio et al., 2012). Therefore, investigating the relationship between self and affect in young adults with hypomanic experiences (BPSD phenotype) may help elucidate mechanisms underpinning psychopathology prior to its full development and in the absence of active illness confounders. This can aid development of early psychological interventions much needed for this younger age group.

A healthy self-identity is flexible and adaptable to environmental changes, including variations in affect (e.g. Bonanno et al., 2004; Kashdan and Rottenberg, 2010). Whilst perceptions of the self vary and are influenced by present contingencies, an adaptive characteristic of human nature is the ability to project oneself into the future with an optimistic self-bias (Weinstein, 1980). The self is not a unitary structure; it comprises various self-related processes and conceptions (Markus and Kunda, 1986), including self-images relating to the present (e.g. I am hard-working) and future (e.g. I will be rich). Furthermore, whilst some self-conceptions are malleable and context-dependent, others are more stable and consistently accessible (Markus and Kunda, 1986). Stable and persistent negative self-images (e.g. I am a failure) are thought to play a role in the maintenance and relapse of depressive states (e.g. Beck, 1967), both in terms of the presence of negative current self-images, which stay rigid regardless of current affect, and fewer positive future self-images.

BPSD is characterised by negative self-related processing (Mansell and Scott, 2006; Whitney et al., 2012) including in vulnerable samples (Lardi Robyn et al., 2012), as well as by self-descriptors linked to high goal-attainment and hypomania (Lee et al., 2010). This hyper-positive sense of self has also been associated with greater relapse after CBT (Lam et al., 2005). Previous work has demonstrated the relevance of examining self-images in psychopathology (e.g. Bennouna-Greene et al., 2012; Jobson and O’Kearney, 2008) and the strong relationship between emotional valence of self-images and well-being in non-clinical samples (Rathbone et al., 2015). Furthermore, it has been argued that BPSD is characterised by an excess of mental imagery (Holmes et al., 2008, 2011) suggesting that investigating self-images may hold particular relevance for this group. However, we know little about the relationship between self-image and affect in BPSD. It is not clear whether, given the mood instability associated with BPSD, the emotional valence (i.e. positive versus negative) of self-images is particularly dependent on mood, or whether other variables play a role (for example, how stable one's self-images are, or the degree of certainty attributed to future self-images).

The aim of the present study was to examine current and future self-image valence, stability over time, and certainty ratings for future self-images and explore how affect and self-image valence, stability and certainty are associated in relation to the bipolar phenotype.

## 2 Method

### 2.1 Participants and procedures

Participants were recruited via advertisements in local newspapers and student groups in the Oxford area based on age (18–50) and scores on the Mood Disorders Questionnaire (MDQ, Hirschfeld et al., 2000) completed online. The MDQ was used to select groups with high versus low hypomanic experience (high=MDQ≥7 versus low=≤3) (Rock et al., 2010), who were then were invited for further assessment at the University Department of Psychiatry, Warneford Hospital, Oxford, UK (study approved by the Research Ethics Committee South Central Oxford B:12/SC/0326). All participants underwent a Structured Clinical Interview for DSM IV-TR (SCID) (First et al., 2002). Exclusion criteria were current or past psychiatric history based on the SCID, any major neurological disorder, and any psychotropic medication. Participants excluded after SCID screening were: 14 participants with high MDQ due to BPSD or past/present depressive episode diagnosis and 4 participants with low MDQ due to past/present depressive episode or eating disorder diagnosis. Eligible participants completed a testing battery (including the measures reported below) immediately after the screening assessment and received reimbursement for their time. One participant failed to complete the full session, resulting in a total sample of 47 participants (66% female, mean age: 23.35, SD=6.07).

### 2.2 Measures

The Mood Disorders Questionnaire (MDQ, Hirschfeld et al., 2000) was used to assess BPSD vulnerability, with cut offs for high and low groups as outlined above. Affect was assessed in terms of depression (QIDS, Rush et al., 2003), anxiety (STAI-S, Spielberger et al., 1983) and mania (ASRM; Altman et al., 1997). Current and future self-images were examined using two open-ended measures of the self (e.g. Rathbone et al., 2008; 2011). Participants completed 10 statements beginning ’I am…’ and 10 beginning ‘I will be…’. All self-images were rated from 0 to 100 for emotional valence (100=very positive) and temporal stability (e.g. ‘How much of the time does this statement describe you?’ or ‘How much of the time in the future might this statement describe you?’ (100=all of the time). I will be statements were also rated for certainty (100=very certain).

### 2.3 Statistical analyses

To test the hypothesis that MDQ group (high versus low) would moderate the relationship between affect (as measured by the QIDS, STAI-state, and ASRM) and self-images, hierarchical multiple regression analyses were performed. Scores on the QIDS, STAI-state and ARSM were entered in the first block, followed by three separate analyses in which the second block examined the interaction between MDQ group and a) QIDS, b) STAI-state, and c) ASRM, respectively. This approach was taken for all regression analyses reported below (only significant results are discussed).

## 3 Results

Mean affect scores and self-image ratings are shown in Table 1. As expected, the high MDQ group reported significantly higher depression (QIDS) and mania (ASRM) scores than the low MDQ group.

All participants reported at least one positive current and future self-image. However, and in line with predictions, 75% of the participants in the high MDQ group generated at least one negative current self-image, compared to 48% of participants in the low MDQ group (*χ*^2^[1, *N*=47]=3.67, *p*=.055). There was no significant difference (*p*=.48) between groups in the proportion of participants generating negative future self-images (13% of low MDQ produced at least one negative future self-image, compared to 21% of high MDQ). There were no significant differences in direct between group comparisons on the self-image subscale measures (Table 1).

First we examine self-image emotional valence. Critically, as shown in Table 2, the QIDS and the STAI-state were significant predictors of self-image valence: the lower the ratings for depression and anxiety, the more positive the self-image score.

Next we examine self-image stability. There were significant moderating effects of MDQ group on the relationship between STAI-state score and ASRM score and stability of negative self-images. This indicates that, for those with high MDQ scores, the relationship between affect and perception of the stability of negative self-images is different compared to those with low MDQ scores.

Finally, certainty ratings for positive future self-images were significantly predicted by the QIDS and ASRM, and there was a significant moderating effect of group on the relationship between ASRM and certainty ratings. Thus, again, there was an effect of BPSD phenotype on the relationship between affect and perceptions of the self in the future.

## 4 Discussion

Results suggest that a bipolar phenotype can shape images of the present and future self in young people. This is the first study, to our knowledge, to examine the valence and stability over time of current and future self-images, and certainty ratings for future self-images, and explore their relationship with affect in a group of young people with and without hypomanic experiences. We showed that the presence of a bipolar phenotype modifies (1) the relationship between affect and the stability of present negative self-images and (2) the relationship between affect and the certainty of positive future self-images; moreover, (3) levels of low mood and anxiety predict the valence of present self-images only, regardless of bipolar phenotype.

The bipolar phenotype predicted the degree of association between affect (both in terms of anxiety and manic features) and the stability of negative current self-images. This suggests that, for young people with hypomanic experiences, there is a different relationship between how anxious or elated they are and how stable their current negative self-images feel, compared to individuals without hypomanic experiences. For example, individuals in the high MDQ group reported negative self-images such as “*most of the time* …I am *shy*, *a worrier*, *quiet*, and *traditional*”, regardless of their affect state. Instead, individuals in the low MDQ group would generate negative self-images such as “I am *often* …*lazy*, *disorganised*, *slow at doing things*” only when presenting with higher anxiety scores. This is consistent with previous evidence of patients with BPSD maintaining a higher number of dysfunctional assumptions about the self after a positive mood induction compared to healthy controls (Lomax and Lam, 2001). A better understanding of this relationship using experimental manipulations may inform psychological treatment improvement. For example, it is possible that cognitive restructuring strategies for BPSD need to intervene directly on the malleability of negative self-images regardless of mood.

Interestingly, BPSD phenotype also predicted the degree of association between elated mood features and certainty ratings about positive future self-images. In those with hypomanic experiences the interaction between elated mood and how certain they feel about positive future self-images was different compared to those without. For example, at similar scores of elated mood a high MDQ participant described their future self-image as “I will *possibly* be *a mother, a wife, famous, able to drive, physically strong, relaxed, a writer and able to cook*”, while a low MDQ participant generated future self-image such as “I will *certainly* be *a graduate, a wife, a mother, a specialised psychologist, career driven, more independent, a role model (for my children)*.” Further investigations of this association may help understand how mania relates to grandiose future projections (or vice versa), consistent with previous data on hyper-positive sense of self (Lee et al., 2010). Previous research highlights biases in future cognitions in BPSD, in particular mental images of the future and their emotional impact (Deeprose et al., 2011; Ivins et al., 2014). Our findings are the first to identify the potential role of certainty estimations. Future studies need to clarify whether BPSD is characterised by feeling increasingly certain about positive self-images becoming true as mood gets ‘high’, or whether greater uncertainty about a positive future self is unaffected by mood state. Alternatively, uncertainty about future self may directly contribute to mood instability. As the transition into mania remains poorly understood, investigating these cognitive mechanisms is key for mania relapse prevention.

As expected, individuals with a BPSD phenotype presented higher levels of both subsyndromal depressive and manic symptoms over the previous week, compared to those without hypomanic experiences. However these differences in affect did not correspond with self-image characteristics. In fact, the valence of current (but not future) self-images was predicted by negative affect regardless of BPSD phenotype. These data indicates that higher levels of low mood and anxiety, even within subsyndromal range, account for rating self-images as more intensely negative and less intensely positive. Instead, the valence ratings of future self-images were not associated with current affect. This suggests that when people imagine themselves in the future they can detach from their current mood, consistent with the role of prospection as a strategy to overcome present difficulties and regulate emotion, fostering hopefulness and optimism (Szpunar et al., 2014). Critically, this self-regulation ability appears to be preserved in young individuals with hypomanic experiences. In fact, while more participants in the BPSD phenotype group (75%) were likely to endorse negative images of the current self compared to the group without hypomanic experiences (less than 50%), almost none – regardless of group – had negative images of the future self.

## 5 Limitations

This was a non-clinical sample of young adults assessed for hypomanic experiences, which limits the extension of this work to clinical levels of psychopathology. This study cannot address the causal relationships between affect, self-images, and BPSD. Future work should use clinical samples and experimental mood manipulation designs in order to address these limitations.

## 6 Conclusions

The self-images we use to define ourselves are closely linked to affect and well-being. BPSD phenotype presents with both alterations and resilience in how self-images and mood shape each other. Further investigations could elucidate how this relationship is affected by illness progression and offer targets for early interventions based on experimental cognitive models.

## Figures and Tables

**Table 1:** Participant demographics and mean self-image valence, stability and certainty subscale scores.

	**Low MDQ (*N*=23)**	**High MDQ (*N*=24)**		
**Scale**	**Mean (SD)**		***t***	**Cohen's d**
Age	23.43 (4.62)	22.46 (5.30)	0.67*	/
Female: Male	15: 8	16: 8	/	/
Years of education	16.22 (2.34)	16.08 (2.70)	0.18	/
QIDS	2.26 (2.05)	6.83 (5.26)	3.96**	−1.03
STAI-S	30.00 (9.53)	35.08 (11.99)	1.61	−0.46
ASRM	1.00 (1.31)	4.17 (2.57)	5.36**	−1.55
Current self-image valence	73.79 (12.68)	66.70 (11.89)	1.98	0.57
Future self-image valence	84.24 (11.30)	82.98 (11.14)	0.39	0.11
Current positive self-image stability	82.55(7.24)	79.03 (10.17)	1.36	0.39
Current negative self-image stability	65.61 (20.63)	67.81 (13.58)	−0.35	−0.12
Future positive self-image stability	78.79 (10.78)	78.04 (10.52)	0.24	0.07
Future negative self-image stability	64.44 (15.03)	58.89 (19.17)	0.42	0.32
Future positive self-image certainty	74.84 (10.76)	72.37 (12.10)	0.74	0.21
Future negative self-image certainty	73.33 (15.28)	62.50 (14.67)	1.03	0.72

Note. Degrees of freedom were 45 for all scores apart from QIDS (30.1) and ASRM (34.6) following correction for equality of variances (Levene's test)

* *p*<.05.

** *p*<.001.

**Table 2:** Hierarchical regression analyses.

Response variable			
Predictor variables	Valence of current self-image	Stability of negative current self-image	Certainty of positive future self-image
Standardised Betas			
Model 1:			
QIDS	−381*	−0.216	−338*
STAI-S	−318*	0.460	−0.193
ASRM	0.197	−0.044	0.304*
Model 2:			
QIDS×Group	0.122	−0.449	−0.204
STAI-S×Group	0.097	−476*	0.065
ASRM×Group	0.033	−719**	0.385*

Note. Group=high MQD versus low MDQ. Grand mean-centred scores were used. All regressions (separate regressions for each potential interaction (a, b, c) included a first step, Model 1, and a second step, Model 2. Model 2 analysed each potential interaction (a, b, c) by adding the interaction term to the Model 1, plus Group, predictors (Model 2). As the purpose of the Model 2 regressions was to investigate potential interactions between Group and Mood, only these regression coefficients are reported here. For valence of current self-image, Model 1: *F*=6.61, *p*=.001; Model 2: QIDS×Group *F*=4.47, *p*=.002; STAI-S×Group *F*=4.48, *p*=.002; ASRM×Group *F*=4.34, *p*=.002; For stability of negative current self-image Model 1 *F*=1.79, *p*=.17; Model 2: QIDS×Group *F*=1.83, *p*=.138; STAI-S×Group *F*=2.72, *p*=.039; ASRM×Group *F*=3.16, *p*=.021; For certainty of positive future self-image Model 1: *F*=3.57, *p*=.020; Model 2: QIDS×Group *F*=2.47, *p*=.045; STAI-S×Group *F*=2.25, *p*=.064; ASRM×Group *F*=3.39, *p*=.011.

* *p*<.05.

** *p*<.01.
